# Frequency of subtype B and F1 dual infection in HIV-1 positive, Brazilian men who have sex with men

**DOI:** 10.1186/1742-4690-9-S1-P31

**Published:** 2012-05-25

**Authors:** Sabri Saeed Sanabani, Ana Carolina Soares de Oliveira, Antonio Charlys da Costa, Mariana Melillo, Sauer Katia Cristina Bassichetto Solange, Maria Santos Oliveira, Priscilla Ramos Costa, Claudia Tomiyama, Helena Tomoko Iwashita Tomiyama, Ester Cerdeira, Sabino Esper, Georges Kal

**Affiliations:** 1 Instituto de Medicina Tropical de São Paulo, São Paulo, Brazil

## Introduction

Co-infection, otherwise known as superinfection, with 2 or more HIV-1 isolates, has been documented frequently. However, in Brazil, few data are available regarding the frequency of HIV-1 co-infection. Because various HIV vaccination studies are in progress, it is important to understand often inter- and intra-subtype co/superinfection occurs in different HIV-infected high risk groups. In this cross-sectional study, we report the frequency of subtype B and F1 co-infection in a clinical group of 41 recently HIV-1 infected men who have sex with men (MSM) in São Paulo, Brazil.

## Material and methods

Proviral HIV-1 DNA was isolated from subject's peripheral blood polymorphonuclear leukocytes that were obtained at the time of enrollment. Each subject was known to be infected with a subtype B virus as determined in a previous study. A small fragment of the integrase gene (nucleotide 4255-4478 of HXB2) was amplified by nested PCR using subclade F1 specific primers. The PCR results were further confirmed by phylogenetic analysis. Viral loads (VL) data were extrapolated from the medical records of each patient.

## Results

In the 41 samples from MSM who were recently infected with subtype B virus, in five patients it was possible to detect subclade F1 proviral DNA, which represents a co-infection rate of 12.2%. In subjects with dual infection the median VL was 5.3 X 104 (range, 1.5 X 104 - 12.5 X 104 copies/ml), whereas in MSM that were infected with only subtype B virus the median VL was 3.8 X 104 copies/ML (range < 400 - 39.3 X 104 copies/ml) (p > 0.8).

## Conclusions

This study indicated that subtype B and F1 co-infection occurs frequently within the HIV-positive MSM population as suggested by large number of BF1 recombinant viruses reported in São Paulo, Brazil. We conclude that the co-infection is a potentially important event that significantly contributes to HIV-1 genetic variability with serious implications for diagnosis, drug treatment and optimal vaccine development.

